# Bilirubin dosage in cord blood: could it predict neonatal hyperbilirubinemia?

**DOI:** 10.1590/S1516-31802004000300005

**Published:** 2004-05-06

**Authors:** Adélia Jeha Nasser Bernaldo, Conceição Aparecida de Mattos Segre

**Keywords:** Newborn, Hyperbilirubinemia, Umbilical cord, Recém-nascido, Hiperbilirrubinemia, Cordão umbilical

## Abstract

**CONTEXT:**

With early discharge, many newborns have to be readmitted to hospital for hyperbilirubinemia to be treated, and this has been held responsible for the reappearance of kernicterus.

**OBJECTIVE:**

To evaluate whether bilirubin levels in cord blood could predict neonatal hyperbilirubinemia that would require treatment, in full-term newborns up to their third day of life.

**TYPE OF STUDY:**

Prospective study.

**SETTING:**

Neonatal Unit of Hospital Israelita Albert Einstein, São Paulo, Brazil.

**PARTICIPANTS:**

380 full-term newborns considered normal: with or without ABO/Rh blood group incompatibility and without other complications.

**PROCEDURES:**

Blood was taken from the umbilical cord for analysis of conjugated, unconjugated and total bilirubin serum levels. The newborns were followed up until discharge, and unconjugated bilirubin that required phototherapy was compared to the cord bilirubin assay. Discriminant analysis was used to classify newborns: with or without risk of needing phototherapy by the third day of life.

**MAIN MEASUREMENTS:**

Bilirubin assay in cord blood; mother's and newborn's blood groups; phototherapy indication.

**RESULTS:**

The mean value for unconjugated bilirubin in cord blood was significantly higher in newborns whose unconjugated bilirubin required photo-therapy. The presence of ABO blood group incompatibility was a significant variable in relation to unconjugated bilirubin that required phototherapy. The most useful cutoff point for unconjugated bilirubin in cord blood was 2.0 mg/100 ml.

**DISCUSSION:**

Cord blood could be collected, stored and used for further analysis of unconjugated bilirubin levels as a means for considering whether or not to discharge a moderately jaundiced child from hospital, in association with other resources.

**CONCLUSIONS:**

Blood incompatibility between mother and child was a predictor for the appearance of hyperbilirubinemia that required treatment. Considering a cutoff point of 2.0 mg/100 ml, it could be concluded that 53% of the newborns who had greater unconjugated bilirubin levels in cord blood would reach levels requiring photo-therapy by the third day of life.

## RESUMO

**CONTEXTO:**

A alta hospitalar precoce vem sendo responsabilizada pelo reaparecimento de casos de kernicterus, ou icterícia nuclear. Assim, pesquisas que visem o encontro de parâmetros que ajudem o neonatologista a prevenir a ocorrência de hiperbilirrubinemia grave são bem-vindas.

**OBJETIVO:**

Avaliar se os níveis de bilirrubina no funículo umbilical podem predizer a hiperbilirrubinemia neonatal que necessita de tratamento em recém-nascidos no termo até o terceiro dia de vida.

**TIPO DE ESTUDO:**

Estudo prospectivo.

**LOCAL:**

Unidade Neonatal do Hospital Israelita Albert Einstein, São Paulo, Brasil.

**PARTICIPANTES:**

380 recém-nascidos no termo considerados normais, com ou sem incompatibilidade sangüínea materno-fetal ABO/Rh e sem nenhuma outra complicação.

**PRINCIPAIS PROCEDIMENTOS:**

Amostra de sangue do funículo umbilical colhida logo após o nascimento e submetida a dosagem de bilirrubinas totais, conjugada e não conjugada. Esses recém-nascidos foram acompanhados até a alta e a ocorrência de níveis de hiperbilirrubinemia não conjugada indicativa de tratamento foi estudada em relação aos níveis do funículo umbilical. Uma análise discriminante foi utilizada para classificar os recém-nascidos em dois grupos, com e sem risco de necessitarem de fototerapia até o terceiro dia de vida (usando o programa Statistical Package for Social Science, versão 9,0) considerando-se o nível de significância menor que 5%.

**VARIÁVEIS ESTUDADAS:**

Bilirrubinas de sangue de funículo umbilical; grupo sangüíneo materno e do recém-nascido; indicações de fototerapia.

**RESULTADOS:**

O valor médio da bilirrubina não conjugada no sangue do funículo umbilical foi significativamente maior entre os recémnascidos que atingiram níveis séricos de bilirrubina não conjugada indicativos de fototerapia. A presença de incompatibilidade sangüínea materno-fetal foi estatisticamente significativa para a ocorrência de níveis séricos de bilirrubina não conjugada indicativos de fototerapia, até o terceiro dia de vida. O ponto de corte de bilirrubina não conjugada do funículo umbilical que se mostrou mais útil foi 2.0 mg/100 ml.

**DISCUSSÃO:**

O sangue do cordão poderia ser coletado e usado para análise dos níveis de bilirrubina conjugada como uma maneira de se considerar ou não a alta de uma criança moderadamente anêmica, em associação com outros recursos.

**CONCLUSÕES:**

A incompatibilidade sangüínea materno-fetal foi preditiva de hiperbilirrubinemia que necessitou tratamento. Quando foi considerado o ponto de corte de 2,0 mg/100 ml no sangue do funículo umbilical, em 53% das vezes, os recém-nascidos atingiram níveis séricos de bilirrubina não conjugada indicativos de fototerapia até o terceiro dia de vida.

## INTRODUCTION

Jaundice is a clinical condition that is often present in pediatric practice and constitutes one of the major issues within the neonatal period. It occurs in both the physiological and pathological processes in newborns.^1-4^ Each year, approximately 60% of the 4 million newborns in the United States are believed to become clinically jaundiced.^5^ Such data is not available for Brazil. At the Neonatal Unit of Hospital Israelita Albert Einstein, 77.6% of all newborns present clinical jaundice and 18.5% need some kind of treatment.^6^

When the newborn stays at the hospital for a 72-hour post-delivery period, it is possible to observe the peaking of the physiological jaundice, thus allowing medical intervention, if necessary. However, in cases of early discharge from the hospital, the newborn may be subject to readmission for phototherapy treatment, because of high levels of unconjugated bilirubin. Such readmission, besides involving extra expenses for both the family and the institution and also exposing a probably healthy newborn to the hospital environment, brings emotional problems and risks to breast-feeding, and is one of the causes of early weaning.^7^

In Brazil, financial pressures have influenced the shortening of hospital stays for mothers and their babies, whether born via vaginal delivery or cesarean section. According to Administrative Rule no. 1016 of the Ministry of Public Health, published in Federal Bulletin no. 167, dated September 1, 1993, full-term healthy newborns should stay in hospital, rooming with their mothers for at least 48 hours.^8^ However, so far no standardized procedure has been laid down in our country either for predicting neonatal jaundice or for following up these early discharged newborns, thus increasing the risk of re-admission and the possibility of brain damage due to kernicterus.^9,10^

Concern regarding early discharge and hyperbilirubinemia in newborns has led to frequent discussions and many controversies.^11-14^ Early hospital discharge has had the implication of reexamining the approach towards neonatal jaundice, now taking into consideration the bilirubin levels presented in the first 24 to 48 hours of life as a means of predicting severe hyperbilirubinemia.^15,16^ Thus, the investigation of parameters that might help the physician prevent the occurrence of severe hyperbilirubinemia is duly justifiable.

The objective of the present study was therefore to verify whether the bilirubin levels found in the cord blood at birth could be indicative of jaundice severity among full-term newborns without complications, up to their third day of life, and also whether mother/child blood group incompatibility could represent a risk factor for such a condition.

## METHODS

This was a prospective cohort study, carried out at the Neonatal Unit of Hospital Israelita Albert Einstein, during the period from September to December of 1997.

### Participants

The initial sample comprised 400 newborns, under the following inclusion criteria: sequentially born, from any type of delivery, both genders, gestational age of over 37 weeks measured by Capurro's method,^17^ birth weight of over 2,500 g and Apgar scores of over 7 at the first and fifth minutes of life, with or without blood group incompatibility between mother and child (ABO or Rh).

The exclusion criteria were: low birth weight or gestational age of less than 37 weeks and any complication arising during the hospital stay that could aggravate the hyperbilirubinemia in these newborns. Thus, 19 cases were excluded from the 400 in the initial sample. Out of the remaining 381 babies, it was necessary to exclude one more, due to possible misinterpretation of the results that would be obtained if this newborn were analyzed together with the group. This newborn had B-O blood group incompatibility, presented an unconjugated bilirubin level in the cord blood of 5.6 mg/100 ml and significant clinical jaundice within the first 24 hours of life. This infant required early phototherapy and also exchange transfusion.

The final sample therefore comprised 380 newborns.

## PROCEDURES

### Experimental Outline

A prospective cohort study model was adopted, in which the group of newborns with the common characteristics previously mentioned was followed up clinically and by laboratory investigation during the period of their hospital stay. At the time of this study, the routine adopted by the neonatal unit involved an observational 72-hour post-delivery period, prior to discharge.

The inclusion of the newborns in the study was done after receiving written consent from their parents or those responsible for them. The study was approved by the Research Ethics Committee of the Teaching and Research Institute of the Hospital Israelita Albert Einstein.

### Experimental Steps

Cord blood samples were collected from all newborns that complied with the protocol inclusion criteria. The samples were sent to the Clinical Laboratory of Hospital Israelita Albert Einstein for the assaying of total, unconjugated and conjugated bilirubin levels.

From then on, the newborns were followed up according to the neonatal unit admission routine, during the 3-day period of their stay. Thus, they were submitted to daily physical examination, carried out by members of the unit's medical team and, whenever necessary, further laboratory tests for bilirubin levels were done, according to the evaluation of the Kramer dermal zones.^18^ In such cases, the recommendation for phototherapy followed the schedule proposed by the Kentucky University Medical Center, which was being used by the unit at that time.^19^

The next step consisted of individual data compilation from the newborn's medical records, for further analysis.

### Main measurements

Neonatal (or total) bilirubin, conjugated bilirubin and unconjugated bilirubin were obtained via the end point colorimeter method, using the Kodak Ektachem 500 device, with a specific method for measuring the neonatal bilirubin levels. Establishment of the mother's and newborn's blood groups was followed by the direct and indirect Coombs test, and also study of the blood compatibility by identification of anti-A and anti-B serum and eluate antibodies, for cases in which this was recommended.

### Statistical planning

The statistical analysis was performed by means of:

- Representation of qualitative variables (category distribution) as absolute (n) and relative (%) frequency values;
- Representation of quantitative variables (continuous) as mean, standard deviation, median, minimum and maximum values;
- Analysis of the association between the phototherapy recommendation due to hyperbilirubinemia and the qualitative variables (category distribution) using the chi-squared test (χ^2^);
- Analysis of differences between groups with and without hyperbilirubinemia indicating phototherapy, with regard to the quantitative variables (continuous) using Student's t test;
- Discriminant analysis to classify the newborns into two groups, according to whether phototherapy was required or not.

The analysis was carried out using the Statistical Package for the Social Sciences (SPSS) program for Windows (SPSS Inc, Chicago, version 9.0) and p values with significance of less than 5% were considered statistically significant.

## RESULTS

Table 1 presents descriptions of the sample of 380 newborns according to gender, weight and gestational age, and the Apgar scores for the first and fifth minutes of life. There were equal numbers of males and females. The mean weight, mean gestational age and Apgar scores were in conformity with the inclusion criteria.

**Table 1:** Sample description for 380 newborns according to gender, weight, gestational age and Apgar scores at the 1^st^ and 5^th^ minute of life

Gender	n (%)
Female	190 (50.0)
Male	190 (50.0)
Weight (g)	
Average ± SD	3336.5 ± 408.5
Median	3290
Minimum/Maximum	2530/4620
Gestational age	
Average ± SD	39 w 2 d ± 5 d
Median	39 w 2 d
Minimum/Maximum	37 w/41 w 3 d
Apgar 1^st^ minute	
Median	9
Minimum/Maximum	8/10
Apgar 5^th^ minute	
Median	10
Minimum/Maximum	8/10

SD = standard deviation; w = weeks; d = days.

The predominant blood group among the mothers was the O type, found in 187 cases (49.21%), followed by the A type in 136 cases (35.79%), B type in 43 cases (11.32%) and AB type in 14 cases (3.68%). The predominant blood group among the newborns was also the O type, found in 175 babies (46.05%), followed by the A type in 153 cases (40.26%), B type in 37 cases (9.74%) and AB type in 15 cases (3.95%).

Out of the 380 newborns in the study, 342 (90%) did not show ABO or Rh blood incompatibility with their mothers. Thirty-eight cases (10%) presented incompatibility: 33 cases of O-A (86.84%) and 5 cases of O-B (13.16%). There was no Rh incompatibility among the 380 newborns. There was no significant difference in the level of unconjugated bilirubin in cord blood between children with or without maternal-fetal blood incompatibility: the mean unconjugated bilirubin levels in cord blood were 1.84 ± 0.45 mg/dl and 1.80 ± 0.70 mg/dl, respectively; t = 0.37; p = 0.711.

According to the clinical evaluation, jaundice was present in the majority of cases (n = 287), but bilirubin levels indicative of photo-therapy occurred in only 19.86% of the jaundiced children, as shown in Table 2. There were no cases of jaundice caused by O-B incompatibility indicative of therapy. The variables of weight, gestational age, Apgar scores (one and five minutes) and gender did not show a significant association with the indication of phototherapy.

**Table 2:** Presence of clinical jaundice and phototherapy indication in 287 newborns

Bilirubin levels not indicative of phototherapy n = 230 (80.14%)				Bilirubin levels indicative of phototherapy n = 57 (19.86%)			
*Phys. n%	O-A n%	O-B n%	Rh n%	*Phys. n%	O-A n%	O-B n%	Rh n%
216	11	3	0	43	14	0	0
93.91	4.78	1.30	0.00	75.44	24.56	0.00	0.00

The unconjugated bilirubin levels in cord blood showed a significant association with the presence of jaundice, when analyzed using Student's t test, as follows: the mean unconjugated bilirubin in cord blood for children who developed jaundice was 1.90 ± 0.4 mg/dl, and for those who did not develop jaundice, it was 1.52 ± 0.49 mg/dl; *t* = 7.12; p < 0.001.

Phototherapy was significantly associated with the presence of blood group incompatibility between mother and child (χ^2^ = 16.42; p < 0.001), as well as with the level of unconjugated bilirubin in cord blood: the mean unconjugated bilirubin in cord blood for children who received phototherapy was 2.12 ± 0.46 mg/dl and for those who did not receive phototherapy was 1.75 ± 0.46 mg/dl; *t =* 5.51; p < 0.001. There also was a significant association between unconjugated bilirubin in cord blood and the newborn's bilirubin level (r = 0.22; p = 0.045).

Discriminant analysis considering unconjugated bilirubin in cord blood (UCB) as a variable resulted in the following equation:


Y=−3.888+2.153×UCB, Wilk'lambda=0.93;χ2=28.25;p<0.001

In accordance with this, the newborns were classified into two groups, i.e. requiring phototherapy and not requiring it. The cutoff point for unconjugated bilirubin in cord blood was 2.0 mg/100 ml. In other words, whenever the unconjugated bilirubin level in cord blood was equal to or greater than 2.0 mg/dl, the probability that the newborn would need phototherapy was 53%.

Table 3 shows the probability of an indication of phototherapy for each value of unconjugated bilirubin in cord blood that was equal to or greater than 2.0 mg/100 ml. Thus, when the unconjugated bilirubin in cord blood was 2.5 mg/dl, the probability of needing treatment was 72%; when the level was 3.0 mg/dl, the probability of needing treatment was 86%, and if it was 3.5 mg/dl, the probability went up to 93%.

**Table 3:** Values of unconjugated bilirubin in cord blood (UCB) and the respective probabilities of indication of phototherapy, according to discriminant analysis: Y = −3.888 + 2.153 x UCB, Wilks’ lambda = 0.93; χ^2^ = 28.25; p < 0.001.

Unconjugated bilirubin in cord blood (mg/dl)	Probability of phototherapy (%)
2.0	53
2.1	57
2.2	61
2.3	65
2.4	69
2.5	72
2.6	75
2.7	78
2.8	81
2.9	84
3.0	86
3.1	88
3.2	89
3.3	91
3.4	92
3.5	93

## DISCUSSION

The hospital stay for both the mother and the newborn should be long enough to allow for the identification of early problems and ensure that the family is duly prepared to take care of the child at home.^20^ However, although early discharge from hospital may seem to be safe, the risk of severe hyperbilirubinemia is an issue that still needs to be resolved and requires specific strategies to prevent a high morbidity rate.^21^

Nonetheless, the concerns about early discharge *vis-a-vis* the occurrence of newborn hyperbilirubinemia have become the theme of many discussions. The more tolerant attitude among neonatologists regarding the bilirubin levels indicative of specific treatment for neonatal jaundice, in association with such early discharges, gave rise to the reappearance of kernicterus in the United States, in the 1990s.^10^ Curiously enough, the same authors that participated in the publication of the guidelines of the American Academy of Pediatrics in 1994 are now highlighting that the reappearance of this condition should serve as an alert for drawing up new guidelines.^15^

In 1977, Risemberg et al.^22^ established a correlation between bilirubin levels in the umbilical cord blood and hyperbilirubinemia in newborns with ABO incompatibility. These researchers concluded that newborns presenting levels higher than 4 mg/100 ml were a group at risk of developing severe hyperbilirubinemia and should be followed up and reassessed, since all of them presented bilirubin serum levels that were higher than 16 mg/100 ml between 12 and 36 hours of life. In the present study, phototherapy was significantly associated with the presence of blood group incompatibility between mother and child, as well as with the unconjugated bilirubin level in cord blood. There was also a significant association between the unconjugated bilirubin in cord blood and the newborn's bilirubin level.

In 1986, Rosenfeld^23^ analyzed a group of 108 full-term newborns according to their risk of developing severe hyperbilirubinemia and concluded that babies with an umbilical cord blood bilirubin level of lower than 2 mg/100 ml had a 4% chance of developing significant jaundice, in comparison with a 25% chance presented by the ones with levels higher than 2 mg/100 ml. In addition, the latter group also presented a higher chance of needing to undergo phototherapy.

Knudsen,^24^ in 1989, carried out a study to demonstrate that jaundiced newborns presented higher umbilical cord blood bilirubin levels than newborns without clinical jaundice. In addition, the number of jaundiced newborns undergoing phototherapy was significantly higher when these levels were higher than 2.3 mg/100 ml, in comparison with the number of jaundiced newborns with no need for treatment and whose bilirubin levels were lower than or equal to 2.3 mg/100 ml. This proved the possibility of defining a newborn risk group for developing neonatal hyperbilirubinemia at birth.

In the present study, the most useful cutoff point for the unconjugated bilirubin levels in cord blood was 2.0 mg/100 ml. With this cutoff point, whenever values that were equal to or greater than this were found, the newborn had a probability of more than 50% that phototherapy would be needed.

As speculation, one may consider the umbilical cord blood to be a kind of “file” for the newborn. As such, it could be collected, stored and used for further analysis of unconjugated bilirubin levels, should a slightly or moderately jaundiced child be considered for early discharge from hospital. Such a proposal may therefore constitute an additional predictive method that is available for evaluating the occurrence of severe hyperbilirubinemia by the third day of life. In association with other resources that are already available,^16^ this proposal may help in assuring safer early discharge for these newborns.

## CONCLUSIONS

The present study led to the conclusion that the unconjugated bilirubin levels in cord blood were indicative of the jaundice severity developed by full-term newborns without complications, up to their third day of life. Levels that were equal to or greater than 2 mg/100 ml indicated a 53% probability of the need for further treatment by photo-therapy. In addition, it was also concluded that the presence of mother/child blood group incompatibility was statistically significant for the occurrence of unconjugated bilirubin serum levels that were indicative of phototherapy treatment during the same three-day period.
